# Dexamethasone intravitreous implant vs bevacizumab for central retinal vein occlusion‐related macular oedema: A prospective randomized comparison—Comment

**DOI:** 10.1111/ceo.13483

**Published:** 2019-04-01

**Authors:** Dejia Wen, Xiao‐Rong Li, Ye He, Xinjun Ren, Chuanzhen Zheng

**Affiliations:** ^1^ Department of Vitreo‐Retinal Tianjin Medical University Eye Hospital Tianjin China

1

We read with great interest the randomized controlled trial by Gado and Macky, presenting the outcomes of patients with central vein occlusion treated with Bevacizumab or dexamethasone implant.^1^

Best corrected visual acuity (BCVA) of candidates in this study was measured at a distance of 2 m using Snellen visual acuity charts, which were converted to a logarithmic scale (logMAR) for statistical analysis. They included patients with macular oedema due to central retinal vein occlusion with best corrected visual acuity 0.3 logMAR to counting fingers. However, BCVA in baseline was showed in Figure 1 of both groups are better than 0.3 logMAR. They described significantly increase in BCVA of both drugs at 6 months. Unfortunately, Figure 1 of the Gado's article showed that both groups mean VA significantly worsened when comparing the baseline, dropping to about 0.6 logMAR at this point. Therefore, we want to question the efficacy of these drugs on BCVA. At this point, we sincerely ask the authors perform analyses of the BCVA of these drugs. Thus, we believe that these corrected data of this study will clarify the functional outcome and comparison of these drugs on patients with CRVO‐MO and these information would be a powerful guide for clinical treatment.

## CONFLICT OF INTEREST

None declared.
